# Pan-Cancer Analysis Revealed *SRSF9* as a New Biomarker for Prognosis and Immunotherapy

**DOI:** 10.1155/2022/3477148

**Published:** 2022-01-12

**Authors:** Jinhui Liu, Yuanyuan Wang, Jian Yin, Yan Yang, Rui Geng, Zihang Zhong, Senmiao Ni, Wen Liu, Mulong Du, Hao Yu, Jianling Bai

**Affiliations:** ^1^Department of Gynecology, The First Affiliated Hospital of Nanjing Medical University, Nanjing 210029, Jiangsu, China; ^2^Department of Biostatistics, School of Public Health, Nanjing Medical University, 101 Longmian Avenue, Jiangning District, Nanjing 211166, China; ^3^Department of Diagnosis, The First Clinical Medical College of Nanjing Medical University, Nanjing 210029, Jiangsu Province, China

## Abstract

**Background:**

Serine/arginine-rich splicing factor 9 (*SRSF9*) is one of the members of *SRSF* gene family and related to the tumorigenesis and the progression of tumor. However, whether *SRSF9* has a crucial role across pan-cancer is still unknown.

**Methods:**

In this study, we used public databases, such as The Cancer Genome Atlas (TCGA), Cancer Cell Line Encyclopedia (CCLE), and Genotype-Tissue Expression (GTEx), to analyze *SRSF9* expression level among tumor and normal cells. Survival analysis, K-M plotter, and PrognoScan were used to analyze the prognosis value of SRSF9, regarding to overall survival (OS), disease-specific survival (DSS), disease-free interval (DFI), and progression-free interval (PFI). Moreover, we performed the correlation between *SRSF9* and clinical characteristics (including the outcome of prognosis), as well as molecular events of tumor mutation burden (TMB), microsatellite instability (MSI), immune checkpoint gene, tumor microenvironment (TME), immune infiltrating cells, mismatch repair (MMR) genes, m6A genes, DNA methyltransferases, and neoantigen with bioinformatics methods and TISIDB, TIMER, and Sangerbox websites.

**Results:**

In general, *SRSF9* expression was upregulated in most cancers, such as BLCA, CHOL, and UCEC, which *SRSF9* was associated with short survival and severe progression. In COAD, STAD, and UCEC, SRSF9 expression was positively related to both TMB and MSI. In BRCA, BLCA, ESCA, GBM, HNSC, LUSC, LUAD, OV, PRAD, TGCT, THCA, and UCEC, both immune score and stomal score showed a negative relationship with SRSF9 expression. Immune score showed a positive relationship with SRSF9 expression in LGG. SRSF9 expression had a significant and positive correlation with six types of immune infiltration cells in LGG, KIRC, LIHC, PCPG, PRAD, SKCM, THCA, and THYM, except in LUSC. In LIHC, SRSF9 was highly significant correlated with most immune checkpoint genes. For neoantigens, correlation between SRSF9 and the quantity of neoantigens was significantly positive in some cancer types. SRSF9 was also correlated with MMR genes, m6A genes, and DNA methyltransferases. In the 33 cancer types, gene set enrichment analysis (GSEA) demonstrated that SRSF9 was correlated with multiple functions and signaling pathways.

**Conclusion:**

These findings demonstrated that *SRSF9* may be a new biomarker for the prognosis and immunotherapy in various cancers. As a result, it will be beneficial to provide new therapies for cancer patients, thereby improving the treatment and prognosis of cancer patients.

## 1. Introduction

SR proteins (serine/arginine-rich splicing factor, SRSF) are a family of proteins participated in RNA splicing [1]. The SR proteins essentially participate in almost every step of the regulation of protein synthesis including mRNA transport, splicing, synthesis of the polypeptide, and formation of the translational initiation complex [2]. Although SR proteins predominantly localize in the nucleus and function splicing, numerous findings highlight the SR proteins involving in diverse process such as mRNA nuclear export, maintenance of genomic stability, translation, and oncogenic transformation [3]. Its relationship with many cancers has been noticed, and SRSF1 is the best studied one, which has been revealed overexpressed in various cancers.

*SRSF9* is an essential member in SRSF gene family, and there are some researches focusing on the mechanism of *SRSF9* in tumorigenesis. In the previous study, tumor suppressive miR-1 induces apoptosis through inhibition of *SRSF9* directly in bladder cancer [4]. Besides, another tumor suppressor miR-802 has been demonstrated that miR-802 targets the 3′-untranslated region of *SRSF9* directly and suppresses *SRSF9* expression, thus inhibiting cell proliferation and inducing apoptosis in cervical cancer [5]. Also, the overexpression of *SRSF9* partly abolished the ferroptosis induced by erastin and tumor growth inhibition in colorectal cancer [6]. But the exact explanation for the connection between *SRSF9* expression and tumor proliferation has not been uncovered.

As previous studies indicated, *SRSF9* have various effects in different conditions; thus, it is essential to discover the relationship between *SRSF9* and pan-cancers from a novel perspective. What's more, the pan-cancer information about the role of *SRSF9* expression in various cancer types is still unknown. This present study used pan-cancer analysis to explore the role of *SRSF9* expression in prognosis and immunity in various cancer types with several public databases. We also studied the relationship between *SRSF9* expression and TMB, MSI, clinical characteristics, TME, immune checkpoint genes, immune infiltration cells, MMR genes, and neoantigens. In this study, we hypothesized that *SRSF9* may be responsible for immune activity inhibition in most of pan-caners, indicating as an unfavorable factor with prognostic value.

## 2. Materials and Methods

### 2.1. Identification of the *SRSF9* Expression Difference in Pan-Cancer

Pan-cancer RNA sequencing data, survival data, and clinicopathological characteristics related to 33 cancer types were downloaded from online database UCEC (https://xena.ucsc.edu/), which was originated from TCGA database [7]. The sequencing data of *SRSF9* were also acquired from GTEx Project and Broad Institute CCLE database to analyze the difference between tumor and adjacent normal tissues. To further learn the gene alteration of *SRSF9* in human cancers, we employed cBioPortal database (https://www.cbioportal.org) to retrieve the mutation and copy number variation of *SRSF9* for visualization [8].

### 2.2. Survival Analysis for the Prognosis Value of *SRSF9*

The patients were divided into high-expression and low-expression groups according to the median expression level of *SRSF9*. Kaplan–Meier survival analysis was conducted here to discover the differential survival outcomes between two groups, and the use of univariate Cox regression model was to determine the favorable or unfavorable prognosis value of *SRSF9*, regarding aspects of overall survival (OS), disease-specific survival (DSS), disease-free interval (DFI), and progression-free interval (PFI). The K-M analysis was conducted by R package “survminer” and “survival,” and forest plot from Cox regression was realized through R package “survival” and “forestplot.”

In addition, the relationship between *SRSF9* expression and survival was further verified in Kaplan–Meier plotter (https://kmplot.com/analysis/) [9, 10] and PrognoScan (http://dna00.bio.kyutech.ac.jp/PrognoScan/index.html) [10, 11], involving clinical outcomes like OS, DSS, disease-free survival (DFS), and relapse-free survival (RFS).

### 2.3. The Analysis of SRSF9 Expression in Clinical Characteristics Using TISIDB

TISIDB (http://cis.hku.hk/TISIDB/index.php) is a web platform for tumor immunity analysis, which contains numerous heterogeneous data types [12] from TCGA database. It contents extensive data on tumor immunity. After submitting the name of interested gene, TISIDB provides us the results of correlation between *SRSF9* expression and clinical stage, tumor grade, immune subtypes, and molecular subtypes. The distribution of *SRSF9* expression and the violin plots of its relationship with immune subtypes, molecular subtypes, clinical stage, and tumor grade in human cancers were presented. Spearman correlation test was applied to do the analysis of the correlation between *SRSF9* and clinical characteristics.

### 2.4. Correlation Analysis of SRSF9 Expression in MSI and TMB

TMB refers to the total count of bases per million that are mutated [13]. Microsatellite instability (MSI) is that the microsatellites' length changes on account of the insertion or absence of repeating units; that is, the change of tandem repeat sequence will lead to the instability of microsatellite [14]. TMB and MSI are related to the development of cancers. They are new biomarkers for evaluating the therapeutic efficacy of immune checkpoint inhibitors.

The gene mutation data in 33 cancer types have come from TCGA database in UCSC Xena. Using the downloaded data, the tumor mutation burden of each sample was calculated by Perl language. The correlation of *SRSF9* expression with TMB and MSI was analyzed by Spearman correlation test, and radar maps were drawn by R package “fmsb” [10].

### 2.5. Correlation Analysis of SRSF9 Expression in TME

TME is the microenvironment in which tumor cells develop and live. It contains different elements, such as tumor cells, some immune cells that surround tumor cells, stroma cells, and so on [15]. The number of stromal cells and immune cells in tumor microenvironment influences the development and growth of cancer cells. R package “ESTIMATE” was used to compute the StromalScore, ImmuneScore, and ESTIMATEScore which was the sum of ImmuneScore and StromalScore [10, 16]. Then, we used Spearman correlation analysis in R to analyze the association between *SRSF9* and stromal score, and immune score.

### 2.6. Correlation Analysis between SRSF9 Expression and the Expression of Immune Infiltration Cells

Tumor Immune Evaluation Resource (TIMER) (https://cistrome.shinyapps.io/timer/) is a database which provides a platform for tumor immune infiltration analysis [17]. It mainly calculated the infiltration scores of six immune infiltration cells: CD4 T cell, B cell, CD8 T cell, neutrophil, dendritic cell, and macrophage. We used the “Gene” module in TIMER to analyze the correlation between the expression of *SRSF9* and the immune infiltration levels across multiple cancer types in TCGA database.

### 2.7. Correlation Analysis between SRSF9 and Immune Cells in Routes of Immunization

In the face of tumor, the human body will motivate a certain immune response through a certain immune pathway to activate immune cells to produce antibodies; at last, we can achieve the immune effect. Sangerbox website (http://sangerbox.com) provides a tool to do TCGA analysis. The association between *SRSF9* and immune cells in some routes of immunization (such as activated CD4 T cell and activated B cell) acquired by a number of immune pathways across 33 cancer types was analyzed in “Gene+” module of Sangerbox website using Pearson correlation test.

### 2.8. The Correlation between SRSF9 Expression and the Expression of Immune Checkpoint-Related Genes

Immune checkpoints refer to programmed death receptor and its ligand. They are a group of molecules that expressed on immune cells. The abnormal expression and dysfunction of immune checkpoint molecules can lead to lots of diseases, such as cancers. Tumor cells secrete some substances that activate immune checkpoints, which, once activated, will suppress T-cell immune function and thus survive [18]. We used Pearson correlation test to investigate the correlation between *SRSF9* and 47 immune checkpoint-related genes (such as ADORA2A, BTLA, BTNL2, CD160, and CD200) in 33 cancer types in “Tool” module of Sangerbox website.

### 2.9. Correlation Analysis between SRSF9 Expression and Immune Neoantigen

Neoantigen is a new abnormal protein encoded by tumor cell mutation gene, which is mainly produced by removal mutation, gene point mutation, gene fusion, etc [19–21]. Using the immune activity of new antigens of tumors, the synthesis of new antigen vaccines can be designed according to the mutation of tumor cells, and the patients can be immunized to achieve therapeutic effect [22]. Neoantigens can contribute to track down tumor cells and therefore serve as important targets for tumor immunotherapy [23].

The quantity of newborn antigens was counted in each tumor sample, and we used Spearman correlation test to study the relationship between these gene expressions and antigen numbers in “Tool” module of Sangerbox website.

### 2.10. Immunotherapy Analysis

Immunotherapy analysis can help researchers understand the relationship between gene expression pattern and immunotherapy effect in one tumor. In GSE78220, GSE67501, and IMvigor210 datasets, we compared the differences of gene expression between patients in response and nonresponse groups based on immunotherapy efficacy. The data of the three datasets were downloaded from Gene Expression Omnibus (GEO) Datasets [24–26]. We draw the boxplot of the two groups and presented the *p* value of the comparison with R package “ggplot2.”

### 2.11. Correlation Analysis between SRSF9 Expression and the Expression of DNA Mismatch Repair Gene, m6A-Related Genes, and DNA Methyltransferase

DNA mismatch repair (MMR) is a system for identifying and repairing DNA replication errors [27], for example, erroneous insertion, misincorporation, and deletion of bases, as well as repairing some other forms of DNA damage. It was widely recognized that the deficiency of MMR could lead to microsatellite instability (MSI), related to some human cancers [28, 29]. DNA methylation is a chemical modification to DNA, and its interaction with histone modifications affects the function of genome [30]. m6A (6-methyladenine) is a common type of RNA modifications, which plays a critical role in cancer progression, such as growth and invasion [31]. R package “RColorBrewer” was used to draw the correlation heatmap between *SRSF9* expression and MMR genes (PMS2, EPCAM, MLH1, MSH2, and MSH6), DNA methyltransferases (TRDMT1, DNMT1, DNMT3A, DNMT3B, and DNMT3L), and m6A-related genes (METTL3, METTL14, YTHDC1, YTHDC2, YTHDF1, YTHDF2, WTAP, RBM15, ZC3H13, HNRNPC, FTO, and ALKBH5) in 33 cancer types.

### 2.12. GSEA

GSEA is a method for analyzing what functions or pathways *SRSF9* influence tumor genesis [32]. The Kyoto Encyclopedia of Genes and Genomes (KEGG) database and the gene ontology (GO) were used for the GSEA with the R package “clusterProfiler.”

### 2.13. Statistical Analysis

All analysis was accomplished by R software version 3.6.3 and various R packages. The comparison of SRSF9 between tumor tissues and normal samples was tested by Wilcoxon rank sum test. The association between *SRSF9* expression and some targets of interest was calculated by Pearson correlation test, including TMB, MSI, immune checkpoint genes, MMR genes, M6A genes, DNA methylation genes, and immune infiltration cells in R. *p* value under 0.05 was regarded as significant. R packages including “ggplot2,” “ggpubr,” “limma,” “survival,” “survminer,” “fmsb,” “ggExtra,” “clusterProfiler,” “ESTIMATE,” “RColorBrewer,” “enrichplot,” and “forestplot” were all used for analysis.

## 3. Results

### 3.1. *SRSF9* Expression Pattern in Pan-Cancer

**Figure 1:**
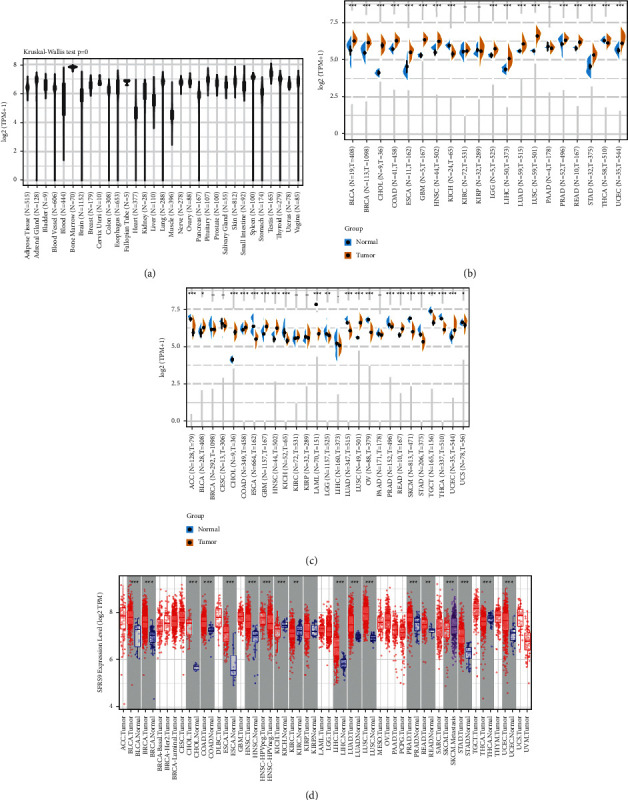
The transcription levels of *SRSF9* in various tissues and tumors. (a) The expression levels of *SRSF9* in different normal tissues from GTEx database. (b) The expression difference of *SRSF9* in tumor and adjacent normal samples in pan-cancer from TCGA database and (c) combining data from TCGA and GTEx database. (d) The expression difference of *SRSF9* in tumor and normal tissues from TCGA database by TIMER. The red color represents the expression of SRSF9 in tumors, and other colors represent the normal tissue.

First, we set out to learn the expression levels of *SRSF9* in different tissues from healthy people with data from GTEx database. As the boxplot shown in Figure 1(a), the *SRSF9* level varied across different types of tissue and was particularly high in bone marrow. For further analysis, the data of *SRSF9* expression in 33 tumor and normal samples were also obtained from TCGA database and we identified the expression differences of *SRSF9* using both the TCGA and GTEx databases. The results are shown in Figures 1(b) and 1(c). Considering TCGA data alone, the difference of expression achieved significance in 17 out of 20 cancer types, with the exception of KIRC, KIRP, and PAAD. And only in THCA, *SRSF9* had increasing expression in adjacent normal tissues instead of tumor samples, which was contrary to the condition in other cancer types. After combining the data from TCGA and GTEx databases, the expression difference turned out to be significant in 21 out of 27 cancer types (exceptions were BRCA, CESC, KIRP, KIRC, LIHC, and PAAD). *SRSF9* was still highly expressed in some tumor types, such as BLCA, CHOL, COAD, GEM, HNSC, LUSC, READ, and UCEC, while in ESCA, KICH, LAML, LUAD, OV, PRAD, SKCM, STAD, TGCT, THCA, and UCS, there were reverse results with significance.

Then, we examined the expression levels of *SRSF9* in pan-cancer using the gene data in TIMER. As Figure 1(d) indicated, the expression of *SRSF9* was significantly higher in tumor samples compared with normal tissues in majority of cancers, including BLCA, BRCA, COAD, CHOL, ESCA, HNSC, LUAD, LIHC, LUSC, STAD, and UCEC. By contrast, the *SRSF9* expression was downregulated in tumor tissues of KICH, KIRC, SKCM, and THCA, which was mainly consistent with the analysis before.

### 3.2. Prognostic Value of *SRSF9* across Cancers

**Figure 2:**
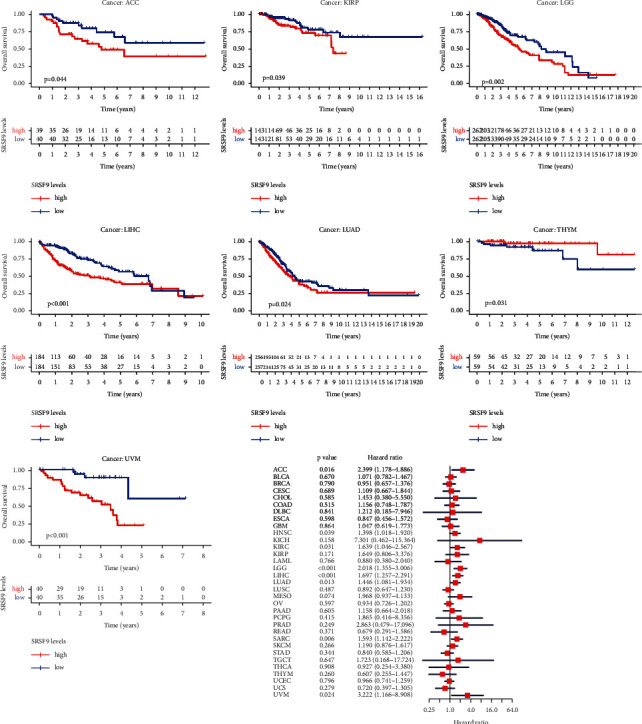
Significant OS difference between high-expression and low-expression groups of *SRSF9* in ACC, KIRP, LGG, LIHC, LUAD, THYM, and UVM and the association between *SRSF9* expression levels with OS in pan-cancer.

To understand the prognostic value of *SRSF9* better in pan-cancer, we studied the relationship between *SRSF9* expression and various kinds of survival outcomes for each cancer, including OS, DFI, PFI, and DSS. In Figure 2, K-M survival curves revealed that high expression of *SRSF9* was apparently associated with poor OS time in ACC, KIRP, LGG, LIHC, LUAD, and UVM, with an exception of THYM, which showed a better prognosis along with the increase of *SRSF9* expression. Specifically, *SRSF9* was seem to be a hazard factor in 8 cancer types: ACC (HR = 2.399, *p*=0.016), HNSC (HR = 1.398, *p*=0.039), KIRC (HR = 1.639, *p*=0.031), LGG (HR = 2.018, *p* < 0.001), LIHC (HR = 1.697, *p* < 0.001), LUAD (HR = 1.446, *p*=0.013), SARC (HR = 1.593, *p*=0.006), and UVM (HR = 3.222, *p*=0.024).

As for DSS results visualized in Supplementary Figure S1, low-expression group significantly survived longer than those in the high expression in 5 cancer types (KIRC, KIRP, LGG, LIHC, and UVM). The univariate Cox regression also displayed that *SRSF9* was related to DSS time in 6 cancer types: ACC (HR = 2.381, *p*=0.019), KIRC (HR = 2.465, *p*=0.001), LGG (HR = 2.338, *p* < 0.001), LIHC (HR = 1.493, *p*=0.023), MESO (HR = 2.603, *p*=0.037), and UVM (HR = 3.482, *p*=0.021). *SRSF9* was all a risk factor in these cancers.

With regard to DFI, Supplementary Figure S2 illustrated that low expression of *SRSF9* was associated with improved OS in many patients of LGG, LIHC, and PCPG, which was opposite in THCA. Cox regression analysis results indicated that *SRSF9* was all a risk factor in LGG (HR = 7.546, *p*=0.002), PCPG (HR = 106.058, *p*=0.043), and PRAD (HR = 5.506, *p*=0.009).

In terms of analysis for PFI displayed in Supplementary Figure S3, we observed high-expressed *SRSF9* correlated negatively with PFI in ACC, KIRC, LGG, LIHC, PCPG, PRAD, and UVM, while related positively with PFI in OV and THCA. Cox proportional hazard model analysis showed that high *SRSF9* expression was associated with unfavorable outcomes in ACC (HR = 2.705, *p*=0.002), KIRC (HR = 1.925, *p*=0.005), LGG (HR = 2.133, *p* < 0.001), LIHC (HR = 1.405, *p*=0.005), PRAD (HR = 2.886, *p*=0.001), and UVM (HR = 7.488, *p* < 0.001), but with optimistic prognosis in OV (HR = 0.778, *p*=0.040).

**Figure 3:**
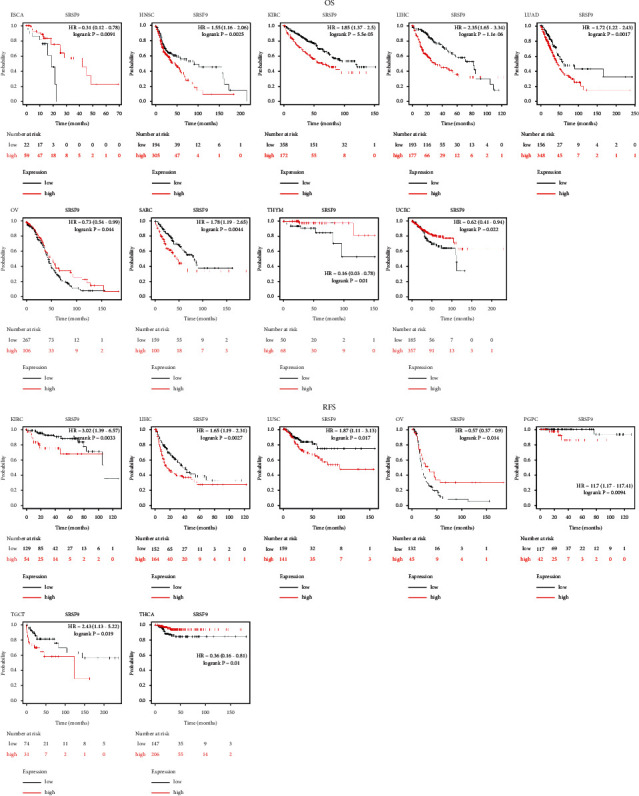
Validation of the relationship between *SRSF9* expression and prognosis in Kaplan–Meier database.

To visualize the impact of *SRSF9* on prognosis in pan-cancer, Kaplan–Meier plotter was employed and it unfolded that overexpression of *SRSF9* had a significant association with poorer overall survival in HNSC, KIRC, LIHC, LUAD, and SARC, and reversely in ESCA, OV, THYM, and UCEC. The correlation with progression-free survival (PFS) was estimated as well, and high levels of *SRSF9* indicated worse PFI in KIRC, LIHC, LUSC, PGPC, and TGCT, except for OV and THCA (Figure 3).

Over and above Kaplan–Meier plotter, we all used the PrognoScan to assess the association between *SRSF9* and prognosis of each cancer. Detailed results are summarized in Table 1. Notably, *SRSF9* played an adverse prognostic role in breast (OS, RFS, DSS, and DFS), lung (OS and RFS), ovarian (OS), blood (OS), skin (OS), and esophagus (OS) cancers. All these evidences suggested that high-expressed *SRSF9* was linked with unfavorable clinical outcomes in majority of cancers, especially in LGG, LIHC, KIRP, and UVM. The exceptions were mainly occurred in OV, THCA, and THYM.

### 3.3. Clinical Stage and Tumor Grade

To investigate the association between *SRSF9* expression and clinicopathological characteristics in various cancers, we assessed *SRSF9* expression with the 33 cancers in stages I, II, III, and IV. Boxplots with significant correlation between gene expression and clinical stage and tumor grade were selected for analysis and interpretation.

**Figure 4:**
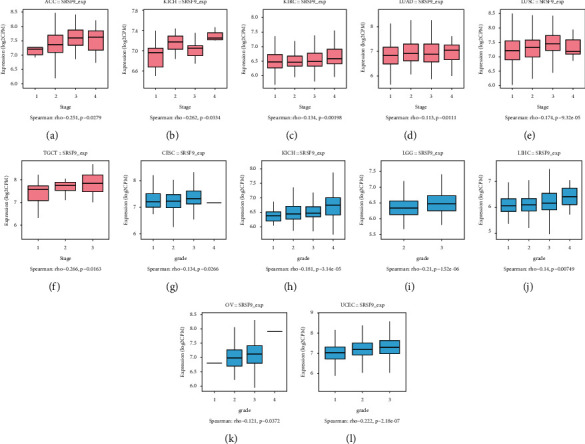
The correlation between *SRSF9* expression and pan-cancer clinical stage using TISIDB: (a) in ACC, (b) in KICH, (c) in KIRC, (d) in LUAD, (e) in TGCT, and (f) in LUSC. The correlation between *SRSF9* expression and pan-cancer tumor grade using TISIDB: (g) in CESC, (h) in KIRC, (i) in LGG, (j) in LIHC, (k) in OV, and (l) in UCEC.

The significant results of the correlation between *SRSF9* expression and clinical stage and tumor grade are shown in Figure 4. In the 33 cancers, the different clinical stages of six tumors showed significant association with *SRSF9*, including ACC (*r* = 0.251, *p* < 0.05), KICH (*r* = 0.262, *p* < 0.05), KIRC (*r* = 0.134, *p* < 0.01), LUAD (*r* = 0.113, *p* < 0.05), TGCT (*r* = 0.266, *p* < 0.05), and LUSC (*r* = 0.174, *p* < 0.001). In ACC, KIRC, LUAD, and TGCT, the expression of *SRSF9* increased as the clinical stage escalated. These results pointed that *SRSF9* expression was associated with disease progression in ACC, KIRC, LUAD, and TGCT. In KICH, *SRSF9* was highly expressed in the stage IV, moderately expressed in the stage II, and lowly expressed in stages I and III. In LUSC, *SRSF9* was highly expressed in stage III, moderately expressed in stages I and II, and lowly expressed in stage IV.

The tumor grade showed significant correlation with *SRSF9* expression in CESC (*r* = 0.134, *p* < 0.05), KIRC (*r* = 0.181, *p* < 0.001), LGG (*r* = 0.21, *p* < 0.001), LIHC (*r* = 0.14, *p* < 0.01), OV (*r* = 0.121, *p* < 0.05), and UCEC (*r* = 0.222, *p* < 0.001). The expression of *SRSF9* increased as the tumor grade escalated in KIRC, LGG, LIHC, OV, and UCEC. That demonstrated that *SRSF9* was a key gene which promotes cancer progression in KIRC, LGG, LIHC, OV, and UCEC. In CESC, *SRSF9* was highly expressed in stage III, moderately expressed in stages I and II, and lowly expressed in stage IV.

### 3.4. Immune Subtype and Molecular Subtype

**Figure 5:**
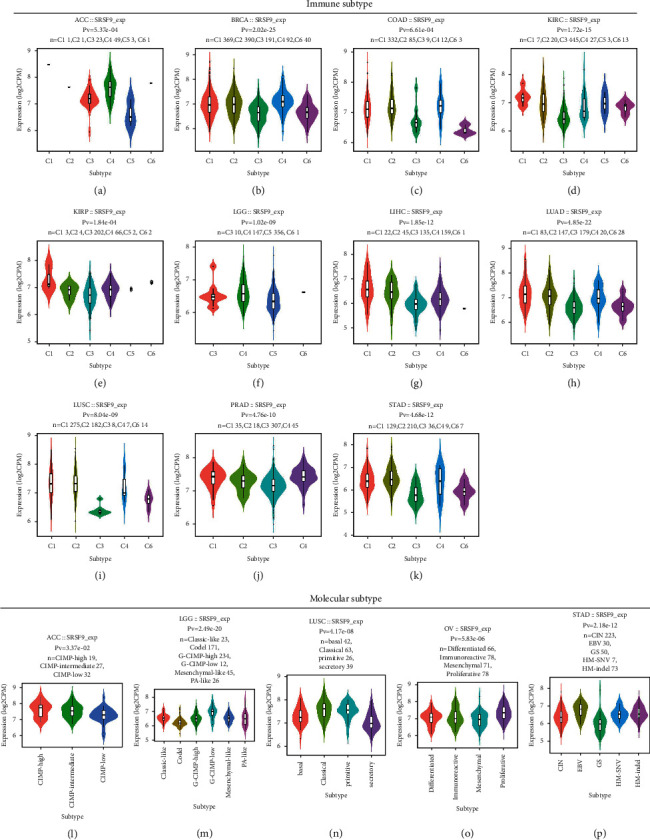
The correlation between *SRSF9* expression and pan-cancer immune subtypes and molecular subtypes using TISIDB: (a) in ACC, (b) in BRCA, (c) in COAD, (d) in KIRC, (e) in LGG, (f) in LUAD, (g) in LUSC, (h) in KIRP, (i) in PRAD, (j) in LIHC, and (k) in STAD. C1 (wound healing); C2 (IFN-gamma dominant); C3 (inflammatory); C4 (lymphocyte depleted); C5 (immunologically quiet); C6 (TGF-b dominant). The correlation between *SRSF9* expression and pan-cancer molecular subtypes using TISIDB: (l) in ACC, (m) in LGG, (n) in LUSC, (o) in OV, and (p) in STAD.

Then, the correlation between *SRSF9* expression and immune subtype and molecular subtype was analyzed (Figure 5). Violin plots with significant correlation between *SRSF9* expression and immune subtypes and molecular subtypes were selected for analysis and interpretation of *SRSF9*'s mechanism.

*SRSF9* expression showed significant correlation to different immune subtypes in ACC (*p*=5.37*e* − 04), BRCA (*p*=2.02*e* − 25), COAD (*p*=6.62*e* − 04), KIRC (*p*=1.72*e* − 15), LGG (*p*=1.02*e* − 09), LUAD (*p*=4.85*e* − 22), LUSC (*p*=8.04*e* − 09), KIRP (*p*=1.84*e* − 04), PRAD (*p*=4.76*e* − 10), LIHC (*p*=1.85*e* − 12), and STAD (*p*=4.68*e* − 12). *SRSF9* was lowly expressed in C3 type in most of the 11 cancers except ACC, CIAD, and LGG. In ACC, COAD, and LGG, *SRSF9* was lowly expressed in C5 type.

The expression of *SRSF9* in different molecular subtypes of ACC (*p* < 0.05), LGG (*p* < 0.001), LUSC (*p* < 0.001), OV (*p* < 0.001), and STAD (*p* < 0.001) was all significantly different. There had no association between *SRSF9* and molecular subtypes been found in other cancers. *SRSF9* was highly expressed in the CIMP-high subtype of ACC, moderately expressed in the CIMP-intermediate subtype of ACC, and lowly expressed in the CIMP-low subtype of ACC. *SRSF9* was highly expressed in the G-CIMP-low subtype of LGG, expressed lowly in the Codel subtype of LGG, and expressed moderately in the classic-like, G-CIMP-high, mesenchymal-like, and PA-like subtype of LGG. The high expression of *SRSF9* was in the primitive and classical subtype of LUSC, the moderate expression of *SRSF9* was in the basal subtype of LUSC, and the low expression of *SRSF9* was in the secretory subtype of LUSC. *SRSF9* was upregulated in the proliferative subtype of OV and downregulated in the mesenchymal subtype of OV. Moreover, *SRSF9* had moderate expression in the differentiated and immunoreactive subtype of OV. *SRSF9* had high expression in the EBV subtype of STAD, moderate expression in the CIN, HM-SNV and HM-indel subtype of STAD, and low expression in the GS subtype of STAD.

### 3.5. TMB and MSI

**Figure 6:**
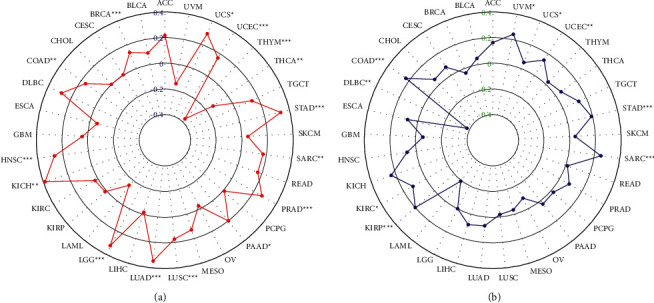
(a) Radar plot showing the correlation between *SRSF9* expression and TMB in pan-cancer. The blue number represents Spearman's correlation coefficient. (b) Radar plot showing the correlation between *SRSF9* expression and MSI in pan-cancer. The green number represents Spearman's correlation coefficient. ^*∗*^*p* < 0.05, ^*∗∗*^*p* < 0.01, ^*∗∗∗*^*p* < 0.001.

The results of the correlation between *SRSF9* and TMB and MSI are displayed in Figure 6. As indicated in Figure 6(a), *SRSF9* expression was significantly (*p* < 0.05) associated with TMB in 15 out of 33 cancer types (BRCA, COAD, HNSC, KICH, LGG, LUAD, LUSC, PAAD, PRAD, SARC, STAD, THCA, THYM, UCEC, and UCS). *SRSF9* expression was negatively related to TMB in THCA and THYM, while it was positively related to TMB in other 13 cancers. KICH had the highest correlation coefficients, and THYM had the lowest one with *SRSF9*. The coefficients indicated that *SRSF9* expression showed a positive correlation with low mutation in THYM and THCA (particularly THYM), but high mutation status in other 13 cancer types.

As presented in Figure 6(b), *SRSF9* expression was significantly (*p* < 0.05) associated with TMB in COAD, DLBC, KIRC, KIRP, SARC, STAD, UCEC, and UVM. *SRSF9* was negatively related to MSI in DLBC and the correlation coefficient was the lowest, while it had a positive relationship with MSI in COAD, KIRC, KIRP, SARC, STAD, UCEC, and UVM. Top 3 correlation coefficients with *SRSF9* levels were in COAD, SARC, and UVM.

In COAD, STAD, and UCEC, *SRSF9* expression was positively related to both TMB and MSI, demonstrating that the higher the expression of *SRSF9* was, the higher the degree of tumor mutation was and the worse condition the cancer was.

### 3.6. Correlation Analysis between *SRSF9* and TME, Immune Infiltration Cells, and Immune-Related Cells in Some Immune Routes

**Figure 7:**
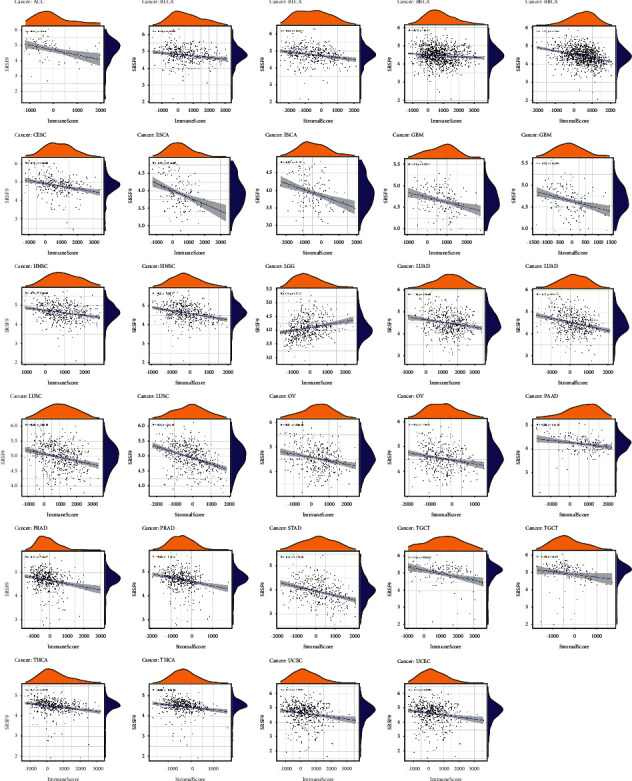
The correlation between *SRSF9* expression and ImmuneScore and StromalScore in pan-cancer.

We measured the correlation between *SRSF9* and immune score and stromal score. Then, we visualized the significant results in Figure 7. As shown in Figure 7, the immune scores in 15 out of 33 cancers showed significant correlation with *SRSF9* expression and the stromal scores in 14 out of 33 cancers had significant correlation with *SRSF9*. In ACC (*r* = −0.43, *p* < 0.05), BLCA (*r* = −0.22, *p* < 0.05), BRCA (*r* = −0.12, *p* < 0.05), CESC (*r* = −0.26, *p* < 0.05), ESCA (*r* = −0.35, *p* < 0.05), GBM (*r* = −0.3, *p* < 0.05), HNSC (*r* = −0.26, *p* < 0.05), LUAD (*r* = −0.18, *p* < 0.05), LUSC (*r* = −0.28, *p* < 0.05), OV (*r* = −0.21, *p* < 0.05), PRAD (*r* = −0.23, *p* < 0.05), TGCT (*r* = −0.29, *p* < 0.05), THCA (*r* = −0.31, *p* < 0.05), and UCEC (*r* = −0.18, *p* < 0.05), immune score was negatively correlated with *SRSF9* expression and the highest correlation coefficient was in ACC. Immune score was positively correlated with *SRSF9* expression in LGG (*r* = 0.25, *p* < 0.001). In BLCA (*r* = −0.26, *p* < 0.001), BRCA (*r* = −0.31, *p* < 0.001), ESCA (*r* = −0.32, *p* < 0.001), GBM (*r* = −0.33, *p* < 0.001), HNSC (*r* = −0.3, *p* < 0.001), LUAD (*r* = −0.27, *p* < 0.001), LUSC (*r* = −0.4, *p* < 0.001), OV (*r* = −0.2, *p* < 0.001), PAAD (*r* = −0.35, *p* < 0.001), PRAD (*r* = −0.25, *p* < 0.001), STAD (*r* = −0.32, *p* < 0.001), TGCT (*r* = −0.36, *p* < 0.001), THCA (*r* = −0.28, *p* < 0.001), and UCEC (*r* = −0.2, *p* < 0.001), stomal scores were negatively correlated with *SRSF9* expression. The higher the expression of *SRSF9*, the more the purity of tumor cells in various cancers.

**Figure 8:**
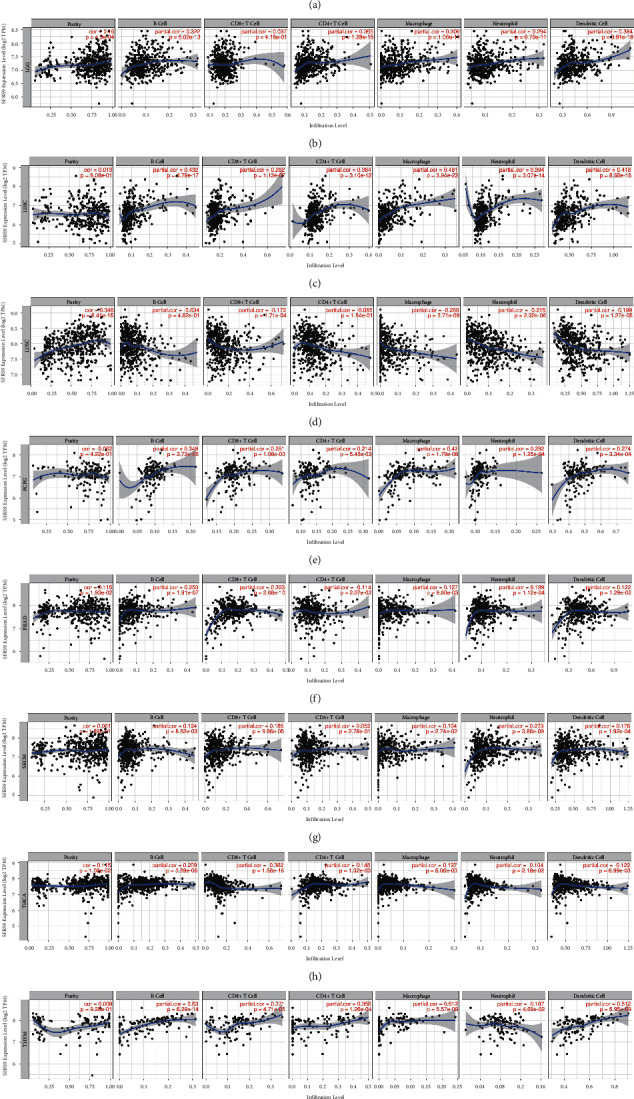
The correlation between *SRSF9* expression and immune infiltrating cells in (a) KIRC, (b) LGG, (c) LIHC, (d) LUSC, (e) PCPG, (f) PRAD, (g) SKCM, (h) THCA, and (i) THYM using TIMER.

Therefore, we studied the association between *SRSF9* and immune infiltrating cells in 33 cancer types from TIMER database, and the significant results given in Figure 8 presented that *SRSF9* was notably correlated with tumor purity and six types of immune infiltrating cells, including CD8+ T cell, CD4+ T cell, B cell, neutrophil, dendritic cell, and macrophage, in KIRC, LGG, LIHC, LUSC, PCPG, PRAD, SKCM, THCA, and THYM. Based on the analysis before, *SRSF9* was related to poor prognosis in some tumors.

Based on the immune analysis results before, *SRSF9* may impact on the immune activities, so we calculated its correlation with immune-related cells. The heatmap shown in Supplementary Figure S4 illustrated that the higher the expression of *SRSF9*, the lower the proportion of immune cells, especially in GBM, OV, LUAD, and so on.

### 3.7. Correlation Analysis between *SRSF9* and Immune Checkpoint Genes and the Number of Immune Neoantigens in Pan-Cancer

**Figure 9:**
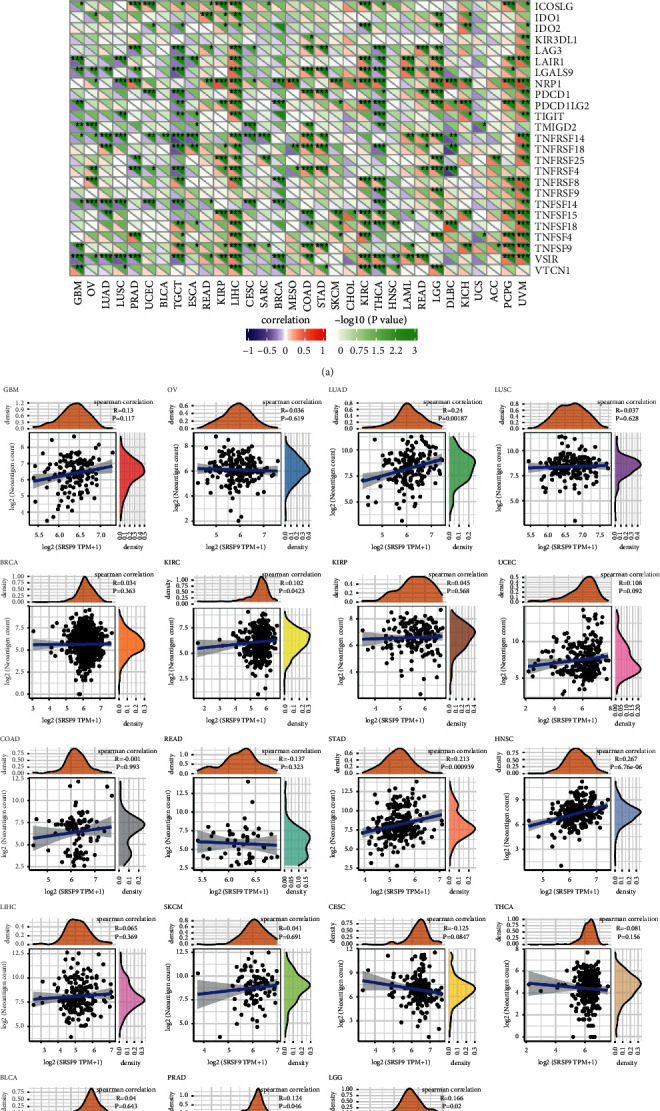
(a) The correlation between *SRSF9* expression and immune checkpoint genes in pan-cancer. Each square corresponded to the correlation between *SRSF9* expression and the expression of one immune checkpoint gene in a particular tumor. The upper triangle of each square represented the magnitude of the *p* value of the correlation test, and the lower triangle represented the magnitude of correlation coefficient (^*∗*^*p* < 0.05, ^*∗∗*^*p* < 0.01, ^*∗∗∗*^*p* < 0.001). (b) The correlation between *SRSF9* expression and the number of neoantigens in pan-cancer.

To analyze whether *SRSF9* expression had a linkage with immune regulation, we analyzed the relationship between *SRSF9* and immune checkpoint gene, neoantigens. Sangerbox was utilized to analyze whether the expression of 47 immune checkpoint genes is correlated with *SRSF9* in 33 cancer types using, and the results are shown in Figure 9(a).

In many tumors, the correlation between *SRSF9* expression and the expression of 47 immune checkpoint genes was found to be significant. CD276 expression was associated positively with *SRSF9* expression in most cancer types. In LIHC, *SRSF9* showed highly significant correlation with the expression of most immune checkpoint genes. There was a positive correlation between *SRSF9* expression and the expression of immune checkpoint-related genes in most tumors, demonstrating that *SRSF9* may provide some help for tumor immunotherapy, thereby benefiting on tumor proliferation.

The results of the correlation between *SRSF9* expression and neoantigens are exhibited in Figure 9(b). As displayed in Figure 9(b), *SRSF9* expression had significantly positive correlation with the number of neoantigens in LUAD (*r* = 0.24, *p*=0.0018), KIRC (*r* = 0.102, *p*=0.042), STAD (*r* = 0.213, *p* < 0.001), HNSC (*r* = 0.267, *p* < 0.001), PRAD (*r* = 0.124, *p*=0.046), and LGG (*r* = 0.166, *p*=0.02). The higher the expression of *SRSF9* was, the greater the number of neoantigens was.

### 3.8. Coexpression of *SRSF9* with Some Specific Genes

MMR gene expression was found positively associated with *SRSF9* in multiple cancers (Supplementary Figure S5(a)). In COAD, LAML, and READ, SRSF9 was negatively related to MMR genes. The relationship between *SRSF9* expression and four DNA methyltransferases was evaluated here, and evidently, *SRSF9* expression was closely related to the expression of these genes across human cancers (Supplementary Figure S5(b)). *SRSF9* was shown to be notably associated with thirteen m6A gene expressions (Supplementary Figure S5(c)).

### 3.9. GSEA

**Figure 10:**
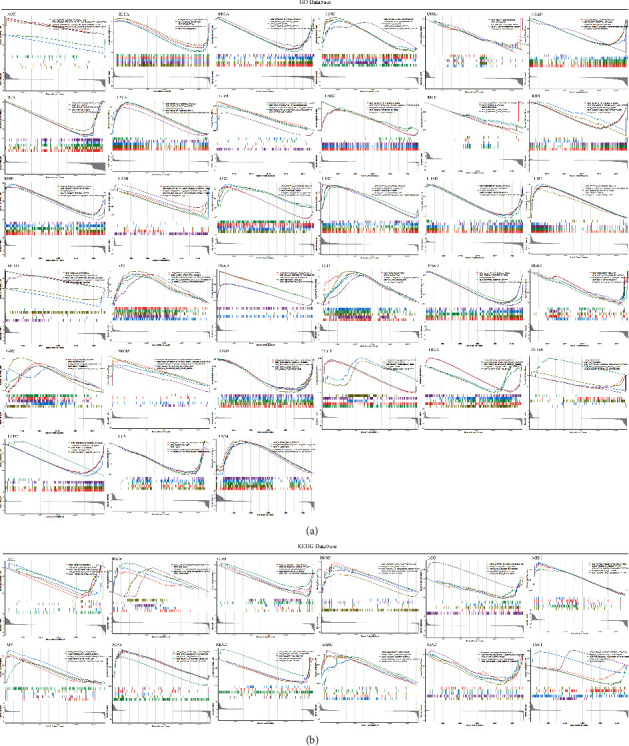
(a) Gene set enrichment analysis of *SRSF9* in the KEGG database in ACC, BLCA, BRCA, CESC, CHOL, COAD, DLBC, ESCA, GBM, HNSC, KICH, KIRC, KIRP, LAML, LGG, LIHC, LUAD, LUSC, MESO, OV, PAAD, PCPG, PRAD, READ, SARC, SKCM, STAD, TGCT, THCA, THYM, UCEC, UCS, and UVM. (b) GSEA enrichment analysis of *SRSF9* with signaling pathways in the KEGG database in ACC, BLCA, GBM, HNSC, LGG, MESO, OV, PCPG, READ, SARC, STAD, and TGCT.

To further investigate the functions or signaling pathways through which *SRSF9* affects tumorigenesis, we used GO (Figure 10(a)) and KEGG (Figure 10(b)) database for gene set enrichment analysis. As displayed in Figure 10(a), in CESC, ESCA, GBM, LGG, HNSC, LIHC, LUSC, PAAD, PCPG, OV, SARC, SKCM, and TGCT, the higher the expression of the *SRSF9* is, the more active these immune functions are. In BLCA, BRCA, COAD, CHOL, DLBC, KICH, KIRP, KIRC, LUAD, LAML, READ, PRAD, STAD, THCA, UCEC, and UCS, the lower the expression of the *SRSF9* is, the more active these immune functions are. In ACC, GO terms were enriched in low *SRSF9* expression, including negative regulation of muscle cell differentiation and negative regulation of vascular associated smooth muscle. GO terms were enriched in high *SRSF9* expression, such as cellular response to cholesterol, negative regulation of cellular response to insulin stimulation, and positive regulation of gluconeogenesis in ACC. In MESO, the defense response to bacterium and positive regulation of myotube differentiation were enriched in low expression of *SRSF9* and chromatin disassembly, NLRP3 inflammasome complex assembly, and DNA packaging complex were enriched in high expression of *SRSF9*. In THYM, protein localization to chromosome centromere was enriched in high expression of *SRSF9* and cornification, keratinization, heme copper terminal oxidase activity, and abnormality of Krebs cycle metabolism were enriched in low expression of *SRSF9*.

As exhibited in Figure 10(b), in BLCA, HNSC, MESO, OV, PCPG, and SARC, the activity degree of *SRSF9*-related signaling pathways was significantly related to the high expression of *SRSF9*. In ACC, GBM, and STAD, the activity degree of *SRSF9*-related signaling pathways was related to the low expression of *SRSF9*. In LGG, olfactory transduction was enriched in high expression of *SRSF9* and amyotrophic lateral sclerosis ALS, calcium signaling pathway, cardiac muscle contraction, and long-term potentiation were enriched in low expression of *SRSF9*. In READ, olfactory transduction was enriched in high expression of *SRSF9* and complement and coagulation cascades, drug metabolism, other enzymes, PPAR signaling pathway, and retinol metabolism were enriched in low expression of *SRSF9*. In TGCT, olfactory transduction and spliceosome were enriched in high expression of *SRSF9* and cytokine-cytokine receptor interaction, drug metabolism cytochrome P450, and metabolism of xenobiotics by cytochrome were enriched in low expression of *SRSF9*.

### 3.10. Immunotherapy

According to the results above, the expression of *SRSF9* was related to immunotherapy. Therefore, we performed immunotherapy analysis on data from the following three databases: GSE78220, GSE67501, and IMvigor210, to investigate whether *SRSF9* expression would affect the effect of immunotherapy. As shown in Supplementary Figure S6, *SRSF9* expression was significantly different between the two groups only in urothelial epithelial tumor, suggesting that *SRSF9* expression in urothelial epithelial tumor affected the effect of immunotherapy. The expression of *SRSF9* was upregulated in the response group, indicating that the higher the *SRSF9* expression, the better the immunotherapy effect patients will have in urothelial epithelial tumor. Trends were observed in Supplementary Figure S6 as patients with low *SRSF9* expression appeared to be more sensitive to immunotherapy in melanoma and renal cancer.

## 4. Discussion

*SRSF9*, also known as SRp30c, belongs to SR family and encodes SR protein which involved in splice site selection in alternative splicing of multiple disease-associated genes, like hnRNP A1 [33], ADAR2 [34], and HPV-16 [35]. Although *SRSF9* has been recognized as an unfavorable factor in several cancers like bladder cancer, cervical cancer, and colorectal cancer, the molecular function and clear mechanism of *SRSF9* in tumorigenesis and proliferation of majority cancers have still been uncovered. Also, there has been no research referring its impact in pan-cancer.

In our recent study, we compared the *SRSF9* expression in normal and tumor tissues of 33 pan-cancers, using the data from several databases for verification, including TCGA, GTEx, and TIMER. These results all showed that *SRSF9* expressed varied in up to 20 types of cancer and was upregulated in a number of tumors, such as BLCA, COAD, CHOL, HNSC, ESCA, LUAD, LIHC, STAD, LUSC, and UCEC. When it comes to the prognostic value of *SRSF9* in pan-cancer, our conclusion was that *SRSF9* was closely connected with survival indicators like OS, DSS, DFI, and PFI. Our survival analysis revealed that high expression of *SRSF9* was clearly linked with poor prognosis in ACC, LGG, LIHC, KIRC, KIRP, PRAD, PCPG, and UVM, with exception for OV, THCA, and THYM. The correlation of its expression with survival time of LIHC, LUAD, OV, PCPG, and THYM was further confirmed by K-M plotter, and it was obvious that *SRSF9* was generally an oncogene in most of the pan-cancer except for several particular tumors such as OV and THYM, which was also supported by results from PrognoScan. In previous studies, *SRSF9* has been reported as a hazard factor for diverse cancers in a lot of studies [3–6, 36], and our current findings in pan-cancer were consistence with this recognition.

Clinical stage, tumor grade, immune subtype, and molecular subtype will reveal the prognostic value of tumor to a certain extent. According to the analysis in TISIDB, *SRSF9* expression was associated with disease progression in ACC, KICH, KIRC, LUAD, LUSC, and TGCT and *SRSF9* was a gene which promotes cancer in KIRC, LGG, LIHC, OV, and UCEC. This is in accord with the conclusion of previous studies [3]; that is, *SRSF9* is a proto-oncogene, and its high expression is related to the genesis and development of tumors. *SRSF9* promotes the multiplication, invasion, and other malignant biological behavior of many cancers. It is reported that the downregulation of *SRSF9* will inhibit cell proliferation in cervical cancer cells [5]. In the present study, *SRSF9* expression showed significant difference in different molecular and immune subtypes in some cancer types. For example, in STAD, *SRSF9* was highly expressed in the EBV subtype of STAD. Therefore, *SRSF9* may be an important biomarker which related to the prognosis of different tumors.

We also studied the correlation between *SRSF9* expression and MSI and TMB. TMB has been proved that they can be a biomarker to effectively predict the efficacy of immunotherapy in multiple cancers [37]. TMB is also a biomarker for the evaluation of ICI therapeutic efficacy, and higher TMB was related to better survival [38]. MSI is also related to prognosis. Some studies showed that high MSI indicates a better prognosis [39, 40]. The present study found that *SRSF9* had a correlation with TMB in 15 cancers and MSI in 8 cancers. In BRCA, COAD, HNSC, KICH, LUAD, LGG, LUSC, PRAD, PAAD, SARC, STAD, UCEC, and UCS, *SRSF9* expression was significantly positive correlated to TMB, demonstrating that the higher the expression of *SRSF9* is, the higher the degree of tumor mutation is. In THCA and THYM, *SRSF9* expression was notably negative correlated to TMB. In COAD, KIRC, KIRP, SARC, STAD, UCEC, and UVM, *SRSF9* expression was significant positive correlated to MSI. In contrast, in DLBC, *SRSF9* expression was significantly negative correlated to MSI. Therefore, *SRSF9* may influence tumorigenesis by involving in the progress of gene alterations. These results also indicated that *SRSF9* seems to be a significant biomarker for the treatment and prognosis in multiple cancers.

Tumor microenvironment is the microenvironment in which tumor cells generate and live. The immune cells and stroma cells in tumor microenvironment can influence the prognosis of cancer and survival outcomes of patients [41]. TME is closely associated with the genesis and metastasis of tumors [42]. Previous study has found that cytokines in tumor microenvironment regulate immune function and ultimately inhibit immune response, leading to tumor progression [43]. Immune cells and stromal cells are included in tumor environment, and they can determine the effect of TME to some extents. In addition, many studies have reported that immune cells are significantly related to the development and progression of tumors. Therefore, the analysis of the components in TME contributes to the development of target drugs for tumor immunotherapy. Our study found that in various cancers, *SRSF9* expression had a negative correlation with the content of immune cell and stromal cell in tumor microenvironment. However, in LGG, the content of immune cell was positively correlated with *SRSF9* expression. Immune infiltration cells play a significant role in tumor microenvironment. Some studies have reported that different degrees of immune infiltration are associated with prognosis and tumor progression [44]. The immune infiltration cells, such as CD8+ T cell, B cell, CD4+ T cell, neutrophil, and macrophage, secrete various factors that influence the TME, regulate tumor behaviors, and have anticancer ability. Many researchers have found that tumor immune infiltration is related to cancer prognosis [45]. We found that in KIRC, LGG, LUSC, PCPG, PRAD, SKCM, THCA, and THYM, *SRSF9* expression had significant relationship with six immune infiltration cells and the purity of tumor. For example, in LUSC, *SRSF9* expression had been shown to negatively correlate with the six immune infiltration cells, but positively correlated with purity of tumor. Based on the above conclusions, in most tumors, the higher the expression of *SRSF9*, the higher the tumor purity. This further supports our previous conclusion that *SRSF9* has a carcinogenic effect in most tumors. Immune checkpoint genes, such as CTLA-4 and PD-1, are important targets of immune checkpoint inhibitors in the treatment of various cancers [46]. Nowadays, immune checkpoint inhibitors (ICIs) are the effective anticancer immunotherapy method. ICI achieves immune effect by blocking immune checkpoint pathways [47]. In the present study, *SRSF9* expression had been shown to positively correlate with most of the 47 immune checkpoint genes in most cancers, such as CD276, CD86, CTLA-4, and CD40. The expression of CD276 was significantly positively correlated with *SRSF9* expression in most cancer types. In LIHC, *SRSF9* expression was highly significant correlated with the expression of most immune checkpoint genes. Similar conclusions were also obtained in the literatures; that is, *SRSF9* can regulate the expression of immunosuppressant GR [48]. Therefore, *SRSF9* can be used as a new drug target for immunotherapy against cancer.

These immunological analyses suggest that *SRSF9* is a very important immunotherapeutic and prognostic target. It can combine with known immune checkpoint inhibitors to strengthen immune infiltration and the response to cancer. However, the mechanism of *SRSF9* in a tumor and its relationship with immunity still need further analysis. There are limited data on the relationship between *SRSF9* and immune-related factors, so we need more studies on the mechanism of *SRSF9* in various cancers to corroborate our results.

Based on the above analysis, we know that *SRSF9* is a very important biomarker for tumor immunotherapy. In addition, we selected three tumors and analyzed the relationship between patients' response to immunotherapy and *SRSF9* expression in these three databases: GSE78220, GSE67501, and IMvigor210. In this paper, we concluded that *SRSF9* expression in urothelial epithelial tumor affected the effect of immunotherapy and the higher the expression of *SRSF9* had, the better the immunotherapy effect of patients had in urothelial epithelial tumor. Combined with the information of the difference in gene expression, SRSF9 was significantly overexpressed in BLCA, but the expression in KICH, KIRC, and KIRP was relatively less or similar to that in normal tissues. Therefore, the results of immunotherapy here further indicate that cancer patients with high SRSF9 expression have better immunotherapy effect.

We also analyzed the correlation between *SRSF9* expression and MMR-related genes, m6A gene, and 4 DNA methyltransferases. DNA mismatch repair (MMR) can repair the errors which rise during DNA replication. It was widely known that mismatch repair (MMR) system can repair the replication errors of microsatellites [49], but deficient MMR (dMMR) would result in the microsatellite instability (MSI) [50]. m6A RNA methylation plays a crucial role in the tumorigenesis and progression of tumors [51]. DNA methylation alterations are also related to tumorigenesis [30]. Based on the present study, our conclusion was that *SRSF9* expression showed a positive correlation with MMR-related genes, m6A gene, and 4 DNA methyltransferases in most cancers. In summary, the results proved the *SRSF9*'s function in mediating tumorigenesis by regulating DNA damage, and DNA and RNA methylation. This is consistent with the previous studies [30, 49–51].

According to the results of GSEA, *SRSF9* showed significant correlation with the activation or inhibition of various carcinogenic pathways and immune functions. For example, in MESO, the high expression of *SRSF9* weakens the body's defense response to cancer cells and the positive regulation of myotube differentiation. Therefore, *SRSF9* may be involved in cancer regulation by influencing the regulation of some immune response and function of some cancer cells. However, as the mechanism of *SRSF9* varies in various tumors, this conclusion needs to be further studied in each tumor, so as to understand how *SRSF9* affects immune functions in a specific tumor and which way *SRSF9* accesses to regulate the immune activity.

At present, few studies have showed the immunological role of SRSF9 in pan-cancer. And our study analyzed the relationship between SRSF9 and many immunological aspects in many ways. Different databases were used to cross-validate our findings, and this strengthens our results. Our study investigated that SRSF9 is a key gene with prognostic value in pan-cancer, deserving more researches' focus. The mechanism of SRSF1 on tumorigenesis has been reported in accumulating studies, while the analysis concentrating on SRSF9 was rare. Thus, this multiomics pan-cancer analysis is meaningful to guide more immune-related experiments for SRSF9.

There are several disadvantages in our study. Firstly, although we use a lot of bioinformatic methods to analyze the relationship between SRSF9 and many immunological aspects, there were few experiments to validate our results. Therefore, a specific clinical cohort is needed to further assess the diagnostic and prognostic potential of SRSF9 in many cancers. Secondly, even though we indicated a reasonable explanation for SRSF9's prognostic value, it is still unknown which way SRSF9 accesses to regulate the immune activity, and more detailed researches are indispensable. Moreover, our findings are still waiting for further validation in the context of both molecule and clinical levels. Thirdly, we use data from TCGA database, KM plotter, and PrognoScan to do the prognostic analysis, but the data may not be always the same. Therefore, further analysis should be used to verify our results.

## 5. Conclusion

In summary, our study indicated that *SRSF9* has high expression in various tumors and high-expressed *SRSF9* had an association with poor survival outcome and disease progression. We also demonstrated *SRSF9*'s relationship with the expression of immune infiltration cells, the expression of immune checkpoint genes, TMB, MSI, and other relative immune indicators, inferring that *SRSF9* may impact on the tumor development via immune suppression. Our careful analysis of this study provides insights into the notable strength of *SRSF9* as a prognostic and immunotherapeutic biomarker for pan-cancer in terms of immunology, offering robust and new evidence for potential development of future immunotherapy and diagnostic study. As a result, it will be beneficial to provide new therapies for cancer patients, thereby improving the treatment and prognosis of cancer patients.

## Figures and Tables

**Table 1:** The relationship between *SRSF9* gene expression and the prognosis of different cancers in PrognoScan.

Gene	Dataset	Cancer type	Endpoint	Number	Cox *p* value	HR	95% CI (low-high)
*SRSF9*	GSE1456-GPL97	Breast cancer	RFS	159	0.003805	3.91	1.55–9.86
*SRSF9*	GSE4922-GPL96	Breast cancer	DFS	249	0.007491	2.84	1.32–6.09
*SRSF9*	GSE4922-GPL96	Breast cancer	DFS	249	0.007793	2.52	1.28–4.99
*SRSF9*	GSE1456-GPL97	Breast cancer	DSS	159	0.012586	4.00	1.35–11.88
*SRSF9*	GSE31210	Lung cancer	RFS	204	0.027333	2.89	1.13–7.43
*SRSF9*	GSE9891	Ovarian cancer	OS	278	0.030832	1.70	1.05–2.74
*SRSF9*	GSE8970	Blood cancer	OS	34	0.030936	3.98	1.14–13.95
*SRSF9*	GSE1456-GPL96	Breast cancer	DSS	159	0.031512	3.03	1.10–8.32
*SRSF9*	HARVARD-LC	Lung cancer	OS	84	0.033228	2.84	1.09–7.42
*SRSF9*	GSE4922-GPL97	Breast cancer	DFS	249	0.034675	2.05	1.05–4.00
*SRSF9*	GSE19234	Skin cancer	OS	38	0.035679	4.31	1.10–16.84
*SRSF9*	GSE11595	Esophagus cancer	OS	34	0.036602	2.33	1.05–5.15
*SRSF9*	GSE1456-GPL96	Breast cancer	RFS	159	0.039336	2.51	1.05–6.00
*SRSF9*	GSE1456-GPL97	Breast cancer	OS	159	0.043242	2.58	1.03–6.47
*SRSF9*	GSE17710	Lung cancer	RFS	56	0.044264	2.48	1.02–6.02
*SRSF9*	GSE3494-GPL96	Breast cancer	DSS	236	0.046057	2.40	1.02–5.66

## Data Availability

The data we used to support the findings of the study are mentioned in this study.

## Supplementary Materials

Supplementary Materials See Figures S1-S6 in the “Supplemental Figure.pdf” for comprehensive image analysis. Figure S1: significant DSS difference between high-expression and low-expression groups of SRSF9 in KIRC, KIRP, LGG, LIHC, and UVM and the association between SRSF9 expression levels with DSS in pan-cancer. Figure S2: significant DFI difference between high-expression and low-expression groups of SRSF9 in LGG, LIHC, PCPG, and THCA and the association between SRSF9 expression levels with DFI in pan-cancer. Figure S3: significant PFI difference between high-expression and low-expression groups of SRSF9 in ACC, KIRC, LGG, LIHC, OV, PCPG, PRAD, THCA, and UVM and the association between SRSF9 expression levels with PFI in pan-cancer. Figure S4: the heatmaps of association between SRSF9 expression and immune-related cells in some immune routes. Figure S5: the heatmaps of association between SRSF9 expression and (a) MMR-related genes, (b) DNA methylation genes, and (c) m6A genes. Figure S6: immunotherapy analysis of (a) melanoma, (b) renal cancer, and (c) urothelial epithelial tumor on data from (a) GSE78220, (b) GSE67501, and (c) IMvigor210 database.
