# Supplementary material for: Pan-Cancer Analysis Revealed SRSF9 as a New Biomarker for Prognosis and Immunotherapy
Source: J Oncol. 2022 Jan 12;2022:3477148. doi: 10.1155/2022/3477148 (PMC8769850; doi:10.1155/2022/3477148)
Supplement: Supplementary Materials — See Figures S1-S6 in the “Supplemental Figure.pdf” for comprehensive image analysis. Figure S1: significant DSS difference between high-expression and low-expression groups of SRSF9 in KIRC, KIRP, LGG, LIHC, and UVM and the association between SRSF9 expression levels with DSS in pan-cancer. Figure S2: significant DFI difference between high-expression and low-expression groups of SRSF9 in LGG, LIHC, PCPG, and THCA and the association between SRSF9 expression levels with DFI in pan-cancer. Figure S3: significant PFI difference between high-expression and low-expression groups of SRSF9 in ACC, KIRC, LGG, LIHC, OV, PCPG, PRAD, THCA, and UVM and the association between SRSF9 expression levels with PFI in pan-cancer. Figure S4: the heatmaps of association between SRSF9 expression and immune-related cells in some immune routes. Figure S5: the heatmaps of association between SRSF9 expression and (a) MMR-related genes, (b) DNA methylation genes, and (c) m6A genes. Figure S6: immunotherapy analysis of (a) melanoma, (b) renal cancer, and (c) urothelial epithelial tumor on data from (a) GSE78220, (b) GSE67501, and (c) IMvigor210 database. [file 3477148.f1.pdf]

Supplemental figures

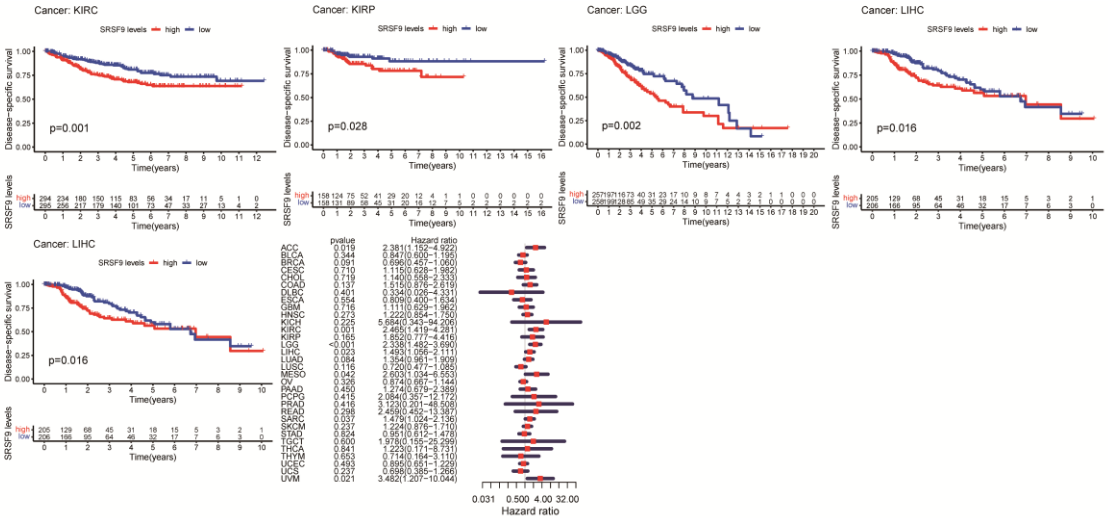

Figure S1: Significant DSS difference between high-expression and low-expression groups of SRSF9 in KIRC, KIRP, LGG, LIHC, UVM and the association between SRSF9 expression levels with DSS in pan-cancer.

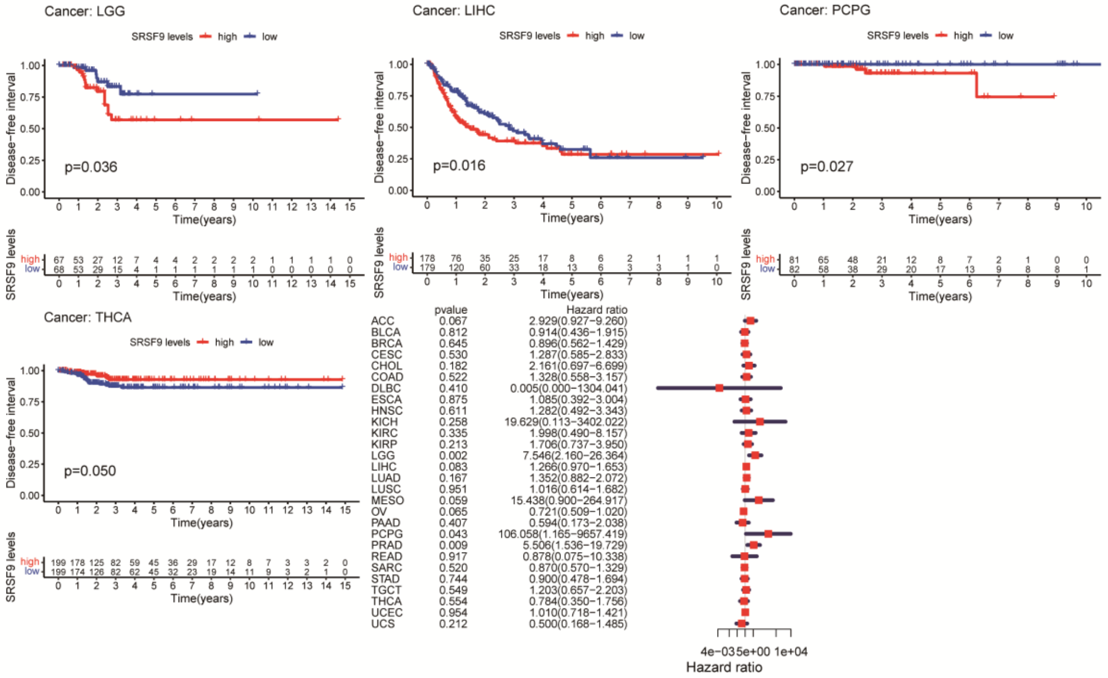

Figure S2: Significant DFI difference between high-expression and low-expression groups of SRSF9 in LGG, LIHC, PCPG, THCA and the association between SRSF9 expression levels with DFI in pan-cancer.

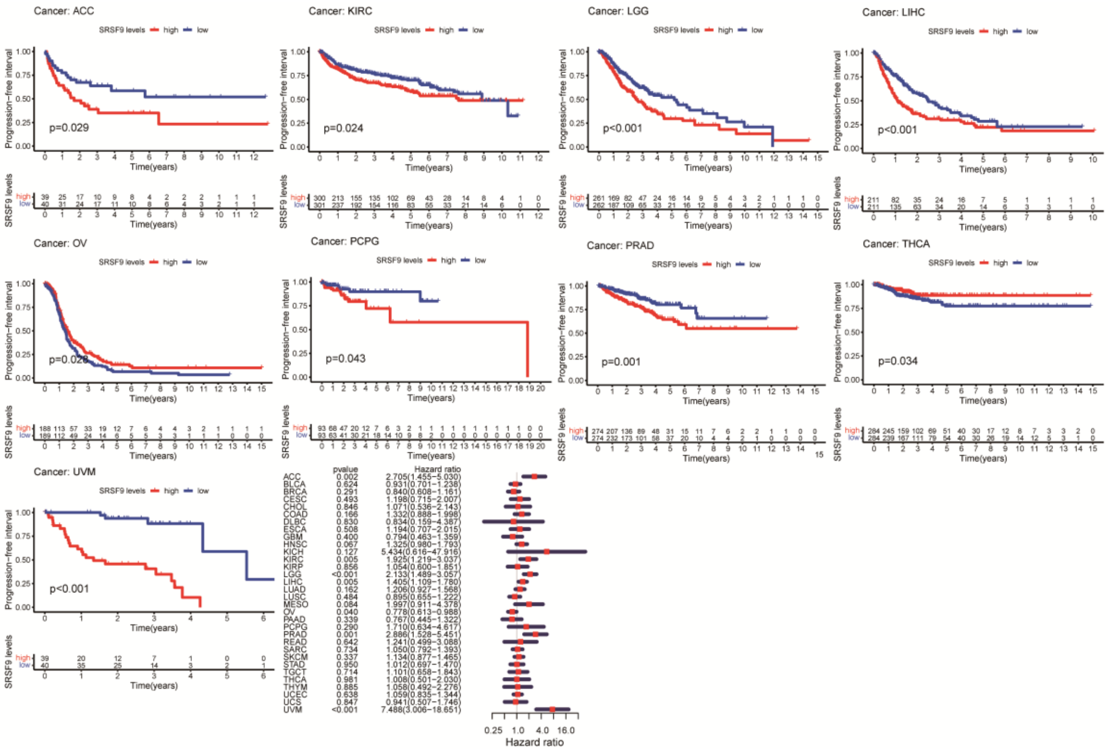

Figure S3: Significant PFI difference between high-expression and low-expression groups of SRSF9 in ACC, KIRC, LGG, LIHC, OV, PCPG, PRAD, THCA, UVM and the association between SRSF9 expression levels with PFI in pan-cancer.

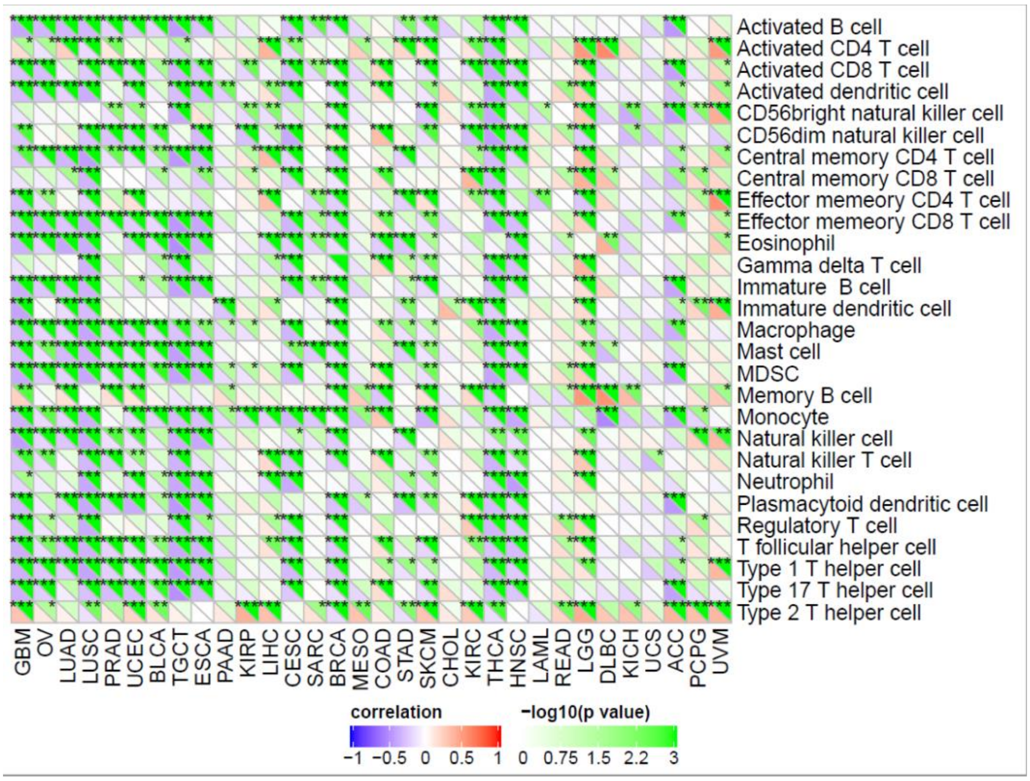

Figure S4: The heatmaps of association between SRSF9 expression and immune related cells in some immune routes.

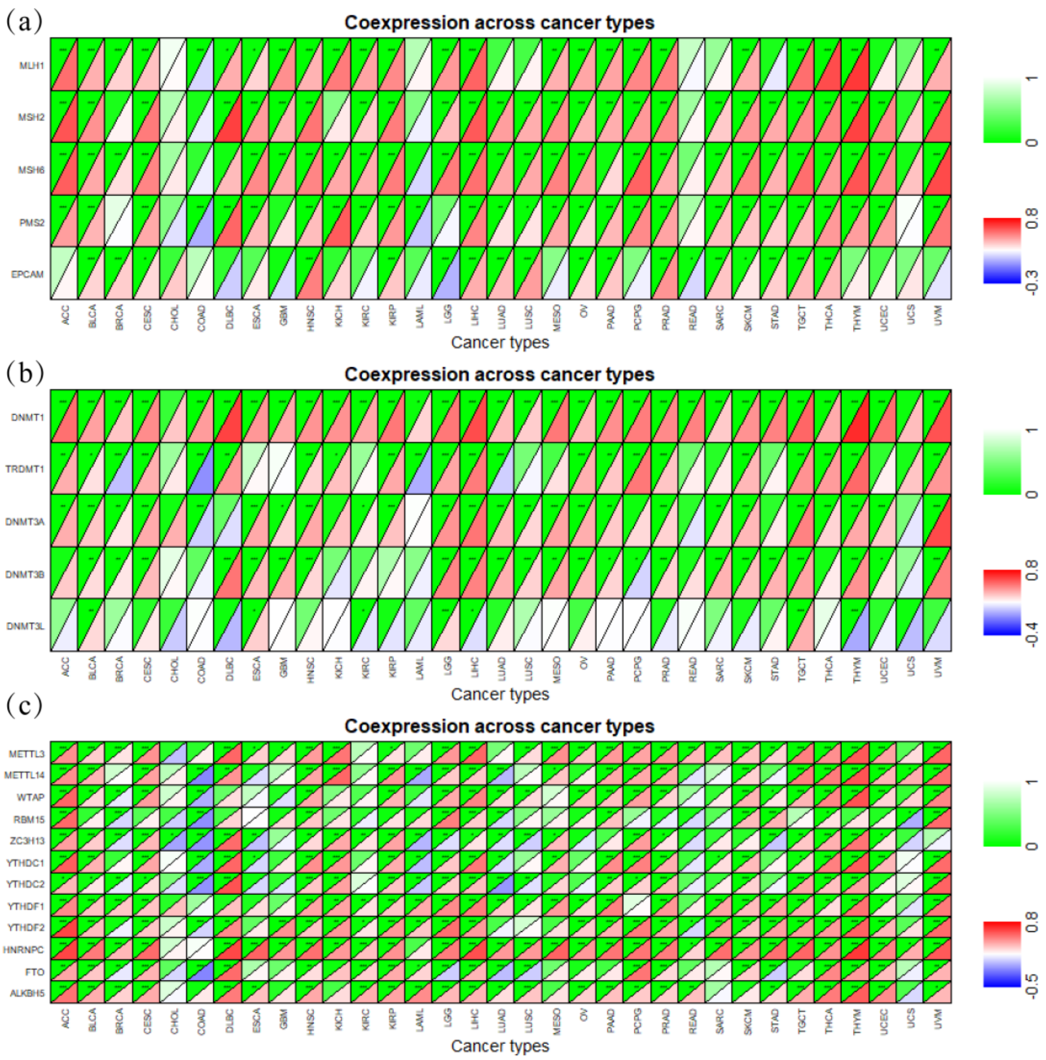

Figure S5: The heatmaps of association between SRSF9 expression and (a) MMR-related genes, (b) DNA methylation genes, (c) m6A genes.

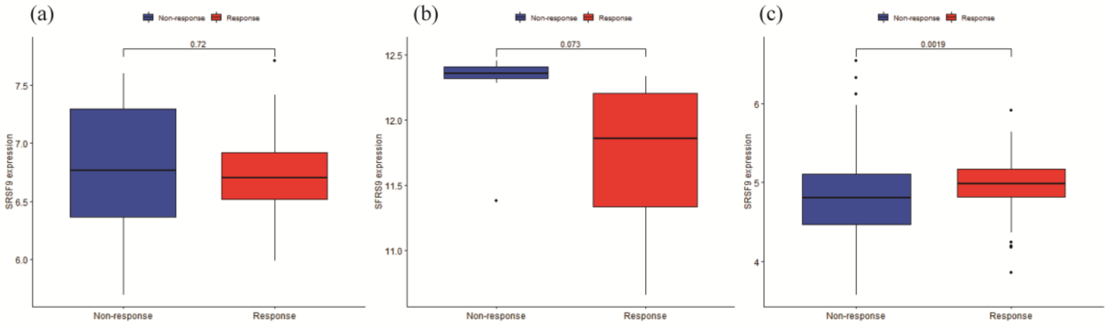

Figure S6: Immunotherapy analysis of (a) melanoma, (b) renal cancer and (c) urothelial epithelial tumor on data from (a) GSE78220, (b) GSE67501 and (c) IMvigor210 database.
